# Enzymatic production of all fourteen partially acetylated chitosan tetramers using different chitin deacetylases acting in forward or reverse mode

**DOI:** 10.1038/s41598-017-17950-6

**Published:** 2017-12-18

**Authors:** Lea Hembach, Stefan Cord-Landwehr, Bruno M. Moerschbacher

**Affiliations:** Institut für Biologie und Biotechnologie der Pflanzen, Westfälische Wilhelms-Universität Münster, Schlossplatz 8, 48143 Münster, Germany

**Keywords:** Amino sugars, Hydrolases, Chromatography, Mass spectrometry

## Abstract

Some of the most abundant biomolecules on earth are the polysaccharides chitin and chitosan of which especially the oligomeric fractions have been extensively studied regarding their biological activities. However, most of these studies have not been able to assess the activity of a single, defined, partially acetylated chitosan oligosaccharide (paCOS). Instead, they have typically analyzed chemically produced, rather poorly characterized mixtures, at best with a single, defined degree of polymerization (DP) and a known average degree of acetylation (DA), as no pure and well-defined paCOS are currently available. We here present data on the enzymatic production of all 14 possible partially acetylated chitosan tetramers, out of which four were purified (>95%) regarding DP, DA, and pattern of acetylation (PA). We used bacterial, fungal, and viral chitin deacetylases (CDAs), either to partially deacetylate the chitin tetramer; or to partially re-*N-*acetylate the glucosamine tetramer. Both reactions proceeded with surprisingly strong and enzyme-specific regio-specificity. These pure and fully defined chitosans will allow to investigate the particular influence of DP, DA, and PA on the biological activities of chitosans, improving our basic understanding of their modes of action, e.g. their molecular perception by patter recognition receptors, but also increasing their usefulness in industrial applications.

## Introduction

As people and industries become more concerned with finding sustainable, natural solutions to modern issues, the use of natural compounds, such as the polysaccharides chitin and chitosan, have skyrocketed in various industries, particularly in agriculture and medicine. Chitin, one of the most abundant biopolymers on earth, is a highly water-insoluble unbranched polysaccharide consisting of β-1,4-linked *N-*acetyl-D-glucosamine units (GlcNAc, A)^1^. It is often processed into a more bioactive derivative called chitosan, a family of molecules generated by partial or complete deacetylation of the C2-bound *N-*acetyl groups of chitin’s GlcNAc units to yield d-glucosamine units (GlcN, D). As the primary amino groups all over the chitosan chain become protonated under slightly acidic conditions (pKa ~ 6.3), chitosans are soluble in weak acids^2^. As the only known naturally occurring polycationic biopolymers, chitosans easily interact with various biomolecules such as most proteins, nucleic acids, and phospholipids^3^. Partially acetylated chitosans, with their part cationic, part hydrophobic character, are even better suited for specific interactions with part anionic, part hydrophobic target biomolecules than the fully deacetylated poly-glucosamine. These properties—along with their biocompatibility and non-toxicity—make chitosans extremely valuable compounds for applications in biomedicine^4,5^, agriculture^6,7^, cosmetics, and the food industry^8,9^.

In the last decade, interest has shifted from the difficult to handle and polydisperse polymers to the more easily soluble oligomers of chitin (COS) and partially acetylated chitosans (paCOS) which can also be fully analysed^10–12^. For these oligomers, their degree of polymerization (DP) as well as their degree of acetylation (DA) are known to strongly influence biological activities^13–16^. For example, Winkler *et al*. recently found that in *Arabidopsis thaliana*, COS with a DP of four (chitin tetramers) are involved in the induction and/or upregulation of genes related to development, vegetative growth, and carbon and nitrogen metabolism^17^. Other studies have shown that COS with higher DPs possess strong elicitor activities towards plant cells (hexamers and larger)^18^; or mimic the molecular dialogue between plants and fungi^19^.

However, when taking a closer look at nearly all studies published dealing with the biological activities of COS and, particularly, paCOS, very few have used pure and well-defined oligomers; instead, broad mixtures have most often been used. These mixtures are typically produced by partial chemical or enzymatic depolymerisation of chitin or chitosan polymers which themselves are often only poorly characterised^17–19^. These processes result in mixtures of which usually at most the average DA and DP is determined and reported. They are sometimes further fractionated according to the DP using size exclusion chromatography (SEC)^20^, but never according to their DA - though mixtures of paCOS with different DAs can and have been produced by using chitosan polymers of different DA as a starting material^21^. Hence, the influence of DP and, in particular, DA on biological activities of paCOS has so far been studied only superficially. Finally, the pattern of acetylation (PA) —another characteristic of chitosans believed to strongly influence biological activities^13,22,23^ — is almost entirely unexplored, mostly because there are no known methods to separate isomeric paCOS, i.e. paCOS with identical DP and DA, but different PAs, and because protocols for the organic synthesis of defined paCOS are still in their infancies^21,24^.

Using such mixtures when investigating biological activities of chitins and chitosans will likely cause the results of such studies to vary and thus not be adequately reproducible, as some of the oligomers in the mixtures may have a certain biological effect while others do not. It is also likely that some oligomers in a mixture will inhibit the biological activities of others, by for example blocking specific receptors, thereby resulting in no detected bioactivity for an oligomer mixture even though some oligomers present possess that activity. A striking example of such a case is the chitosan octamer with an alternate sequence of GlcN and GlcNAc units, DADADADA (GlcN*-*β1,4-GlcNAc)_4_, which inhibits the elicitor activity of the fully acetylated chitin octamer by binding to the chitin receptor, but preventing its chitin-induced dimerization and, thus, activation^23^. Though experimental evidence for the hypothesis is missing owing to a lack of the required isomeric chitosan octamers, inhibitor activity is thought to depend on the alternating PA of the paCOS used which possesses one face with four exposed, hydrophobic acetyl groups able to bind the receptor, but which exposes four charged free amino groups on the opposite face, preventing binding of a second receptor molecule. Clearly, fully defined paCOS are urgently required to further our understanding of structure-function relationships of the biological activities of partially acetylated chitosans.

The most promising approach towards the production of fully defined paCOS is the use of regio-selective chitin deacetylases (CDA, EC 3.5.1.41) for the partial deacetylation of chitin oligomers of defined DP^22,25–32^. The best-known example for such a CDA is NodB from Rhizobium bacteria which specifically deacetylates the non-reducing end unit of a chitin oligomer A_n_ (n = DP) leading to the mono-deacetylated paCOS DA_(n−1)_^25^. More recently, *Vc*CDA from *Vibrio cholerae* has been shown to specifically deacetylate the unit next to the non-reducing end of a chitin oligomer, yielding another mono-deacetylated paCOS, ADA_(n−2)_^26^. Interestingly, these bacterial CDAs can also act on each other’s product, so that their combination can yield the double-deacetylated paCOS D_2_A_(n−2)_^33^. A small number of fungal CDAs have also been reported which differ in their regio-selectivity. *Pgt*CDA from the plant pathogenic fungus *Puccinia graminis* f.sp. *tritici* deacetylates all units of chitin oligomers except for the two units closest to the non-reducing end, yielding A_2_D_(n−2)_^30^. *Pes*CDA from the plant endophytic fungus *Pestalotiopsis* spec. in addition leaves the reducing end unit acetylated, yielding A_2_D_(n−3)_A^22^. In contrast, the first characterized fungal CDA, *Cl*CDA from the plant pathogenic fungus *Colletotrichum lindemuthianum*, is able to fully deacetylate chitin oligomers, yielding D_n_. However interestingly, this enzyme has also been used in reverse direction, to *N-*acetylate a fully deacetylated glucosamine oligomer (D_n_), yielding the dimer AD when acting on the glucosamine dimer D2 as a substrate^34^.

In the present work, we have investigated a number of recombinant CDAs from bacterial, fungal, and viral origin to assess their regio-selectivity and their ability for reverse action. Combining enzymatic deacetylation and enzymatic *N*-acetylation, we successfully produced all of the fourteen possible partially acetylated chitosan tetramers, each with not only a defined DA but also a defined PA. Additionally, we present a method of purification that can reach >95% purity, making these defined paCOS suitable for further investigations regarding structure-function relationships of chitosans, but also making them available for direct use in various fields of application.

## Materials and Methods

### Enzymes – Origins, heterologous expression and purification

Chitin deacetylases from different sources (bacterial, viral, and fungal) were expressed in different expression hosts (*E. coli* BL21 (DE3), *E. coli* Rosetta2 (DE3) [pLysRARE2], *E. coli* Lemo21 (DE3), and *Hansenula polymorpha*) (Table 1). The three bacterial CDAs used are NodB from *Rhizobium sp*. GRH2 (NCBI acc. No. AJW76244.1), *Vc*CDA from *Vibrio cholerae* (NCBI acc. No. AAF94439.1), and *Bc*CDA5 from *Bacillus* sp. The viral CDA used in this study (*Cv*CDA) originates from the *Chlorovirus* CVK2. All these CDAs were heterologously expressed in different variants of *E. coli*. The five fungal CDAs used in this study are *Cn*CDA2 (EMBL Nucleotide Sequence Database acc. no. AJ938050) and *Cn*CDA4 (fpd1/d25, Uniprot acc. no. Q96TR5) from *Crytococcus neoformans*, *Pes*CDA from *Pestalotiopsis* spec. (NCBI acc. no. APH81274.1) as well as *Pgt*CDA from *Puccinia graminis* f.sp. tritici (NCBI acc. no. XP_003323413.1) and *Pa*CDA from *Podospora anserine* (NCBI acc. no. CAP60162). Phylograms based on full amino acid sequences but also based on NodB homology domains are shown in Supplementary Figure S1. The fungal CDAs were either heterologously expressed in variants of *E. coli* or *Hansenula polymorpha*. All enzymes were purified using fast protein liquid chromatography (FPLC) according to Hamer *et al*.^35^ and Lim *et al*.^36^, taking advantage of either a HIS6-tag or a strep-tagII attached to the corresponding CDA. This information is summarized in Table 1. Final protein concentrations were determined by either the Bradford^37^ or the bicinchoninic acid (BCA) assay (Pierce BCA Protein Assay Kit- Thermo Fisher Scientific) for protein quantitation.

**Table 1:** Summarized information about the CDAs used in this study, their origin host as well as the chosen expression host and the protein-tag used for purification (FPLC).

Name	Origin	Expression host	Tag	Reference
NodB	*Rhizobium* sp. GRH2	*E. coli* BL21 (DE3)	Strep-tag II	^33^
*Vc*CDA	*Vibrio cholerae*	*E. coli* Rosetta2 (DE3) [pLysRARE2]	Strep-tag II	^33^
*Bc*CDA5	*Bacillus licheniformis*	*E. coli* Rosetta2 (DE3) [pLysRARE2]	HIS6-tag	^40^
*Cv*CDA	CVK2 *Chlorovirus*	*E. coli* Lemo21 (DE3)	Strep-tag II	^41^
*Cn*CDA2	*Cryptococcus neoformans*	*Hansenula polymorpha*	HIS6-tag	^42^
*Cn*CDA4	*Cryptococcus neoformans*, fpd1	*E. coli* Lemo21 (DE3)	Strep-tag II	^42^
*Pes*CDA	*Pestalotiopsis* spec.	*E. coli* Rosetta2 (DE3) [pLysRARE2]	Strep-tag II	^22^
*Pgt*CDA	*Puccinia graminis* f.sp. *tritici*	*E. coli* Rosetta2 (DE3) [pLysRARE2]	Strep-tag II	^30^
*Pa*CDA	*Podospora anserina*	*Hansenula polymorpha*	HIS6-tag	^32^

### Partial deacetylation of the chitin tetramer (A4) using different chitin deacetylases

The chitin tetramer (1 mg/ml) purchased from Seikagaku (Tokyo, Japan, 95% purity) was incubated with 0.8 µg of each enzyme in 22 µl (0.036 mg/ml) for 98 h (37 °C, 50 mM triethanolamine buffer or 50 mM sodium bicarbonate, both pH 7). Reactions were stopped by heat (98 °C, 15 min) and products were analyzed using ultra high performance liquid chromatography-electrospray ionization-mass spectrometry (UHPLC-ESI-MS) as previously described^11,33^.

### Partial N-acetylation of the chitosan tetramer (D4) using different chitin deacetylases

The chitosan tetramer (1 mg/ml) purchased from Biosynth (East Falmouth, Massachusetts, USA, 95% purity) was incubated with 0.8 µg of each enzyme in 22 µl (0.036 mg/ml) for 98 h (37 °C, 2 M sodium acetate, pH 7). Experiments were performed in three independent repetitions, reactions were stopped by heat (98 °C, 15 min), and final products were analyzed using UHPLC-ESI-MS^11,33^, as briefly described below.

### UHPLC-ESI-MS analysis of chitin (COS) and chitosan oligomers (paCOS)

COS/paCOS were analyzed according to Hamer *et al*.^33^ using a Dionex Ultimate 3000RS UHPLC system (Thermo Scientific, Milford, USA) coupled to an ESI-MS^n^ detector (amaZon speed, Bruker, Bremen, Germany). Separation of different (pa)COS was performed using hydrophilic interaction chromatography (HILIC). A combination of a VanGuard pre-column (1.7 µm, 2.1 × 35 mm) with an Acquity UHPLC BEH Amide column (1.7 µm, 2.1 × 150 mm; both purchased from Waters Corporation, Milford, MA, USA) was used. (Pa)COS-separation was performed by using a gradient of solvent A (80% acetonitrile, 20% water) and B (20% acetonitrile, 80% water), both containing 10 mM NH_4_HCO_2_ and 0.1% (v/v) formic acid. For product analysis, the column oven temperature was set to 75 °C and the flow rate to 0.8 ml/min. The separation of samples was done over 5.5 min using the following elution profile: 0–0.8 min, isocratic 100% A; 0.8–3.3 min, linear from 0% to 70% (v/v) B; followed by column re-equilibration: 3.3–4.3 min linear from 70% to 0% (v/v) B, then from 4.3–5.5 min isocratic 100% (v/v) A. MS-detection was performed in a positive mode and mass spectra were acquired over a scan range of m/z 50–2000 using the enhanced resolution scan mode, and were analyzed using Data Analysis 4.1 software (Bruker, Bremen, Germany). Quantification of (pa)COS present in different samples were obtained by quantifying peak areas for single masses – known to correspond to certain defined (pa)COS - in comparison to controls of either the chitin or the chitosan tetramer treated the same but without adding enzyme

### Determination of the pattern of acetylation of paCOS

About 10 µg of each sample was used for determining the pattern of acetylation according to Cord-Landwehr *et al*.^11^. The reducing end of paCOS was labeled using H_2_^18^O in two steps (1 × 5 µl, 1 × 10 µl, 70 °C). Separation of samples was done as described above, having the target mass set to 840 (m/z). Isolation was done for m/z 791.32, 749.31, 707.30 with an isolation width of 1.0 m/z; fragmentation was carried out using a gradient of the amplitude of 80 to 120% over 20 ms. MS/MS spectra were acquired over a scan range of m/z 50–2000 and were also analyzed using Data Analysis 4.1 software (Bruker, Bremen, Germany).

### Medium-scale production and purification of paCOS

The production of the paCOS GlcN*-*GlcN*-*GlcNAc-GlcN (DDAD) was scaled up to 1.6 mg. As such, 12 µg of *Pes*CDA and 4.5 µg of *Cn*CDA4 were used over 24 h of incubation at 37 °C in 2 M sodium acetate buffer, pH 7. Products were again analyzed using UHPLC-ESI-MS as described above^11,33^. Production of the other three mono-acetylated and further purified paCOS was performed by using different CDAs during either deacetylation of the GlcNAc tetramer (ADDD: *Vc*CDA and *Pgt*CDA, DADD: NodB and *Pgt*CDA) or *N*-acetylation of the GlcN tetramer (DDDA: NodB, *Vc*CDA, and *Pes*CDA).

Using the Dionex Ultimate 3000RS UHPLC system with a 250-nm UV detector (Thermo Scientific, Milford, USA) in combination with a semi-preparative column (Luna 5 µm CN 100 A, 250 × 10 mm, Phenomenex, Torrance, California, USA), the tetramer DDAD was separated from buffer-components and paCOS of different DA. PaCOS-separation was performed using a gradient of solvent A (80% acetonitrile, 20% water) and B (20% acetonitrile, 80% water), both containing 10 mM NH_4_HCO_2_ and 0.1% (v/v) formic acid. For product separation, the column oven temperature was set to 50 °C and the flow rate to 2 ml/min. Separation of samples was performed over 51 minutes using the following elution profile: 0–10 min, isocratic 100% A; 10–40 min, linear from 0% to 75% (v/v) B; followed by column re-equilibration: 40–41 min linear from 75% to 0% (v/v) B, then from 41–51 min isocratic 100% (v/v) A. Fractions of different volumes (1–5 ml) were collected and again analyzed regarding (pa)COS present as well as purity using UHPLC-ESI-MS analysis as described above^11,33^. Fractions containing the paCOS of interest (DDAD) were combined. Using a rotary evaporator at 40 °C, acetonitrile was removed; all other volatile compounds were removed by washing and drying samples several times using a rotational vacuum concentrator at 30 °C, followed by lyophilization.

## Results and Discussion

### Enzymatic *N*-acetylation of the glucosamine tetramer

**Figure 1:**
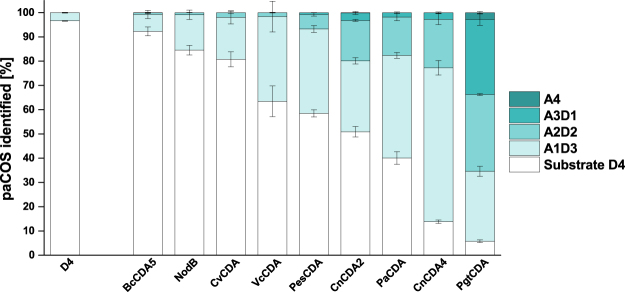
Relative amounts of different chito-oligosaccharides after performing enzymatic *N-*acetylation of the chitosan tetramer (D4, [GlcN]_4_) using different CDAs [*Bc*CDA5, NodB, *Cv*CDA, *Vc*CDA, *Pes*CDA, *Cn*CDA2, *Pa*CDA, *Cn*CDA4, *Pgt*CDA] for 98 h in 2 M sodium acetate buffer, pH 7. Data summarized in this figure have been generated by semi-quantitative HILIC-ESI-MS analysis and performed in triplicate; standard deviations are given by grey bars.

In this study, the ability of nine different CDAs to *N-*acetylate chitosan oligomers—the reverse of their natural function of deacetylation—was investigated using the fully deacetylated glucosamine tetramer as a substrate. We used three bacterial CDAs (NodB from *Rhizobium* sp. GRH2, *Vc*CDA from *Vibrio cholerae*, *Bc*CDA5 from *Bacillus licheniformis*), five fungal CDAs (*Cn*CDA2 and *Cn*CDA4 from *Cryptococcus neoformans*, *Pes*CDA *from Pestalotiopsis* spec., *Pgt*CDA from *Puccinia graminis* f. sp. *tritici, Pa*CDA from *Podospora anserina*) as well as one viral CDA (*Cv*CDA from *Chlorovirus* CVK2), which were all heterologously expressed in either *E. coli* or *H. polymorpha* and purified using affinity chromatography. Equal amounts (mass) of each CDA were incubated with a commercially purchased chitosan oligomer (DA 0%, DP 4) under acetate-enriched conditions (2 M). As the aim was to investigate the overall ability of each CDA to *N-*acetylate the chitosan and not its maximum specificity under given conditions, relatively high amounts of each CDA (enzyme, E) were incubated with the substrate (S) in a ratio (E/S) of 1 to 25 for 100 h at 37 °C, knowingly accepting a possible loss of regio-specificity. The relative amounts of chitosan tetramers after performing *N*-acetylation of the glucosamine tetramer (Fig. 1) were determined through separation and analysis using UHPLC-ESI-MS. The relative amount of different chito-oligosaccharides after performing enzymatic deacetylation of the chitin tetramer in different, individually suitable buffers is shown in Supplementary Figure S2.

All CDAs proved active in reverse catalysis, partially *N-*acetylating the glucosamine tetramer under the given conditions (Fig. 1). Clearly, remarkable differences in the total amount of product produced after 98 hours between different CDAs were visible regarding the extent of *N*-acetylation and, consequently, the DA of the products. The bacterial and viral CDAs clearly resulted in less *N-*acetylated products than the fungal CDAs. The lowest total amount of products formed after 98 h was measured for *Bc*CDA5, seven times lower than for the bacterial CDA which formed the most products under these conditions, namely *Vc*CDA, and forty times lower than for the most strongly product-forming fungal CDA under these conditions, namely *Pgt*CDA. Consequently, the non-fungal CDAs mostly produced mono-acetylated tetramers after incubation for 98 h at 37 °C, while all fungal CDAs led to products of which two, three or sometimes even all four GlcN residues were *N-*acetylated. To better compare the total amount of products formed as well as the regio-specificity of the CDAs during de- and *N-*acetylation, the same total amounts (mass) of enzymes were used for both *N-*acetylation of D4 and deacetylation of A4. In the next sections, we will discuss regio-specificity of the nine CDAs tested during de- and *N-*acetylation.

### Regio-specificity during deacetylation and *N*-acetylation in comparison

**Figure 2:**
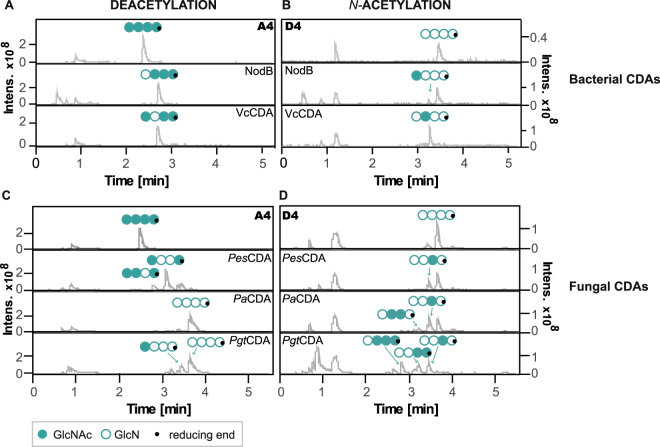
Base peak chromatograms of HILIC-ESI-MS analysis showing products after enzymatic deacetylation of the fully acetylated GlcNAc tetramer (A4, [GlcNAc]_4_, filled circles) (**A,C**) and after enzymatic *N-*acetylation of the fully deacetylated GlcN tetramer (D4, [GlcN]_4,_ open circles) (**B,D**) using the bacterial CDAs NodB and *Vc*CDA (**A,B**) and the fungal CDAs *Pes*CDA, *Pa*CDA and *Pgt*CDA (**C,D**).

To investigate the enzymes’ regio-specificity during both de- and *N-*acetylation, we carried out quantitative analysis of the PA by performing ^18^O-labelling of the reducing ends of all products followed by MS/MS analysis according to Cord-Landwehr *et al*.^11^. Figure 2 shows the results for a selection of bacterial (A,B) and fungal (C,D) CDAs for deacetylation (A,C) and *N-*acetylation (B,D). The figure shows base peak chromatograms of HILIC-ESI-MS analysis and the paCOS representing the most commonly occurring PA for each product, as determined by MS/MS analysis.

For deacetylation, Fig. 2A and C show the COS produced by deacetylation of A4 using different, already well-characterized CDAs, including as concerns the PA of their products. Specifically, NodB was shown to remove the acetyl group from the GlcNAc residue at the non-reducing end of the chitin tetramer, whereas *Vc*CDA deacetylated the GlcNAc unit next to the non*-*reducing end—two regio-specificities already well described in literature^25,26^. In contrast, regarding deacetylation performed by fungal CDAs, all three fungal CDAs shown in Fig. 2 are known to first deacetylate the chitin tetramer at the GlcNAc unit next to the reducing end and then either stop (*Pes*CDA^22^), continue to deacetylate the reducing end (*Pgt*CDA^30^), or deacetylate all four GlcNAc residues in a stepwise fashion (*Pa*CDA^32^). Regarding this deacetylation pattern, these three fungal CDAs are highly specific when using ideal enzyme-substrate ratios, but they are known to partially lose specificity when reaction conditions change^38^. The previously described PA generated by these three fungal CDAs can be observed for *Pa*CDA, but not for *Pes*CDA and *Pgt*CDA, as mainly either the double-deacetylated paCOS ADDA (*Pes*CDA) or, in the case of *Pgt*CDA, the fully deacetylated glucosamine tetramer as well as the mono-acetylated paCOS ADDD were observed (Fig. 2C). These differences in the observed versus expected PA probably occurred because these enzymes were used in excess, as the equal amounts used of each CDA resulted in there being ca. nine times the ideal amount of *Pes*CDA and ca. two times the ideal amount of *Pgt*CDA.

Regarding the ability of these CDAs to perform *N*-acetylation, Fig. 2B and D show LC-MS spectra as well as the main products of the same CDAs after *N-*acetylation of the glucosamine tetramer (D4). Results show that after 98 h, most of the enzymes tested resulted in a less strong change in DA during *N-*acetylation than during deacetylation, which is probably caused by the prevailing salt concentrations (2 M); however, we found that the regio-specificity was not influenced. Our experiments show that NodB solely attacked the sugar residue of the non-reducing end (yielding DAAA and ADDD), *Vc*CDA only acted on the sugar unit next to the non-reducing end (yielding ADAA and DADD), and *Pes*CDA, *Pa*CDA, and *Pgt*CDA in a first step act on the sugar residue next to the reducing end (yielding AADA and DDAD). *N-*acetylation of the fully deacetylated glucosamine tetramer using *Pgt*CDA under the given conditions additionally resulted in the double *N-*acetylated paCOS DDAA and the triple *N-*acetylated paCOS DAAA. These experiments for the first time demonstrate that a range of different CDAs (bacterial and fungal) are able to *N-*acetylate chitosans, and —even more noteworthy— to do so while keeping their regio-specificity associated with deacetylation of chitin oligomers. This ability offers an excellent new tool for generating well-defined paCOS not only in terms of DP and DA, but also for their PA.

**Figure 3:**
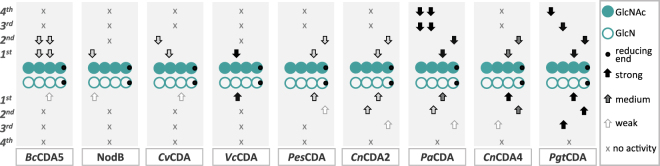
Mode of action of different CDAs (bacterial, viral, and fungal) on the chitin tetramer (A4, [GlcNAc]_4_, filled circles) shown in the upper part, as well as on the chitosan tetramer (D4, [GlcN]_4,_ open circles) in the lower part. The reducing end of the (pa)COS always points to the right and is marked by a black circle. No activity is symbolized by an x, weak activity by an unfilled arrow, medium-strong activity by a grey-filled arrow, and strong activity by a black-filled arrow; arrows point to the unit of the (pa)COS which is preferentially de- or *N-*acetylated during either the 1^st^, 2^nd^, 3^rd^, or 4^th^ attack by the different enzymes. Data summarized in this figure have been generated by (semi-)quantitative HILIC-ESI-MS analysis followed by ^18^O-labelling of the reducing end and MS/MS analysis.

To further generalize the ability of CDAs to *N-*acetylate COS while maintaining their regio-specificities, we directly compared all nine of the CDAs used in this study (bacterial, viral, fungal) for deacetylation as well as *N-*acetylation. The number of positions which have been either de- or *N*-acetylated as well as the main PA generated are presented in Fig. 3 which summarizes the results obtained after 98 h of incubation at 37 °C from three biological replicates of nine CDAs either deacetylating the chitin (AAAA, upper) or *N-*acetylating the fully deacetylated chitosan tetramer (DDDD, lower). Arrows indicate not only the occurrence (“x” if there was no activity observed) but also the main position(s) of the attack.

Our general hypothesis that CDAs maintain their regio-specificity of deacetylation even during *N-*acetylation (which was already shown above for NodB, *Vc*CDA, *Pes*CDA, *Pa*CDA, and *Pgt*CDA) was confirmed by CDAs *Bc*CDA5, *Cn*CDA2, and *Cn*CDA4, which show this behavior in at least their first attack (Fig. 3). *Cv*CDA showed an overall very low amount of products produced by *N-*acetylation, which might be caused by an overall instability of *Cv*CDA under the given salt-rich conditions; thus, it performed very weak and most apparently non-specific *N-*acetylation. As such, this should be further investigated by assessing *Cv*CDA’s stability under different conditions as well as by trying to find more suitable reaction conditions for *N-*acetylation using *Cv*CDA. Nevertheless, we were able to show a general ability of this unique viral enzyme (*Cv*CDA) to *N-*acetylate glucosamine oligomers. Most of the other CDAs tested in this study resulted in complementary PA during deacetylation and *N-*acetylation, even during subsequent attacks of the sugar chain (2^nd^, 3^rd^). These results again strongly indicate that CDAs are at least as specific in generating a defined PA during *N-*acetylation as during deacetylation. This regio-specificity of CDAs is defined by six loops surrounding the active site described in the subsite-capping model of Andres *et al*.^39^. The flexibility as well as the size of these loops control the binding of the substrate in the active site of the enzyme—thereby defining the position of attack, and thus the PA of the oligomers generated. In addition, it is well-described that enzyme-substrate interactions of CDAs are mainly based on stacking interactions between certain aromatic amino acids and the sugar rings itself. Based on this, acetylated GlcNAc and deacetylated GlcN tetramers might bind to the active site in a similar way, so that the enzymatic attack occurs at the same position for both substrates. This would result in a consistent regio-specificity for both deacetylation and *N-*acetylation as shown experimentally in this study.

### Producing well-defined paCOS by combining regio-specific CDAs

**Figure 4:**
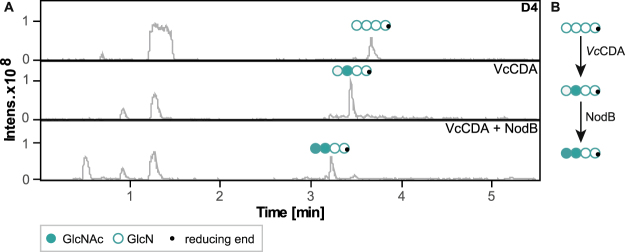
Combination of bacterial CDAs during *N-*acetylation. (**A**) Base peak chromatogram of HILIC-ESI-MS analysis showing products after *N-*acetylation of the chitosan tetramer (D4, [GlcN]_4,_ open circles) by two bacterial CDAs (*Vc*CDA, NodB) yielding in a first step the mono-acetylated paCOS by *Vc*CDA, and in a second step the double-acetylated paCOS by NodB. (**B**) summarizes this production route.

This newly discovered regio-specificity during *N-*acetylation enables us to produce well-defined chitosans and to thereby enlarge the library of available paCOS that have a defined PA—one of the properties of chitosans that is believed to influence various biological activities. Taking a closer look at the deacetylation of COS, it has already been shown that CDAs can be combined to produce paCOS with well-defined DAs and PAs^33^. These authors showed that by combining NodB, alone yielding the mono-deacetylated paCOS DA_n-1_, and *Vc*CDA, alone yielding the mono-deacetylated paCOS ADA_n-2_, the overall product to be the very pure double-deacetylated paCOS D_2_A_n-2_. After showing that most CDA keep their regio-specificity also during *N-*acetylation, we next investigated the enzymes’ abilities to be combined during *N-*acetylation (Fig. 4). Results are only shown for first using *Vc*CDA and then using NodB, but reversing this order resulted in identical products (Supplementary Figure S3). We showed that further incubation of DADD (produced by *Vc*CDA) with NodB resulted in an almost pure product, mainly containing the double-acetylated paCOS AADD (Fig. 4B). This is not a new product to be generated using CDA, as it can also be produced by using *Pgt*CDA in a deacetylation reaction on the chitin tetramer under optimized conditions, but it does support the principle that CDAs in general can be combined during *N-*acetylation of COS.

**Figure 5:**
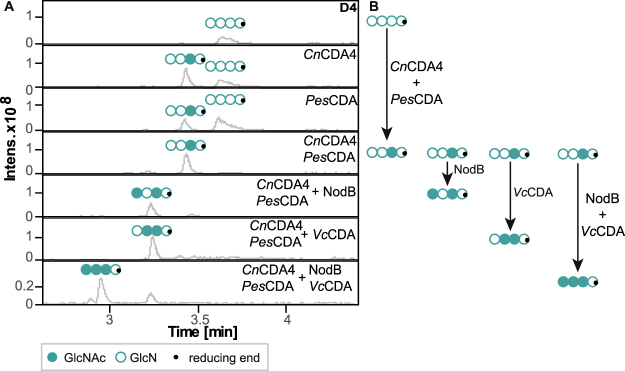
Combination of bacterial and fungal CDAs during *N*-acetylation. (**A**) Base peak chromatograms of HILIC-ESI-MS analysis showing products after *N*-acetylation of the chitosan tetramer (D4, [GlcN]4, open circles) by several bacterial and fungal CDAs in different orders (*Cn*CDA4,* Pes*CDA, *Vc*CDA, NodB), resulting in four different but new single-, double-, and triple-deacetylated products. Figure B summarizes these different production routes.

To determine if this observation held for CDAs from organisms other than bacteria, we next combined the fungal CDAs *Pes*CDA and *Cn*CDA4 (Fig. 5). LC-MS chromatograms of the products that were obtained when *Cn*CDA4 and *Pes*CDA were used, individually and in combination, to *N-*acetylate D4 are shown in Fig. 5A. When using 0.25 µg of *Cn*CDA4 (enzyme-substrate ratio 1:200), around 50% of the D4 was converted into DDAD, whereas when *Pes*CDA was used (0.6 µg, enzyme-substrate ratio 1:83), only 30% was converted into the same product. Surprisingly, when combining both CDAs by using exactly half of the amounts mentioned above, around 90% of the substrate was converted into the mono*-*acetylated paCOS having a highly pure PA of DDAD. This indicates that when both CDAs were combined, there was either a synergistic effect, stabilization, or competition for binding the substrate, thereby making the enzymes more active when being combined. This effect should be investigated further in future studies.

**Figure 6:**
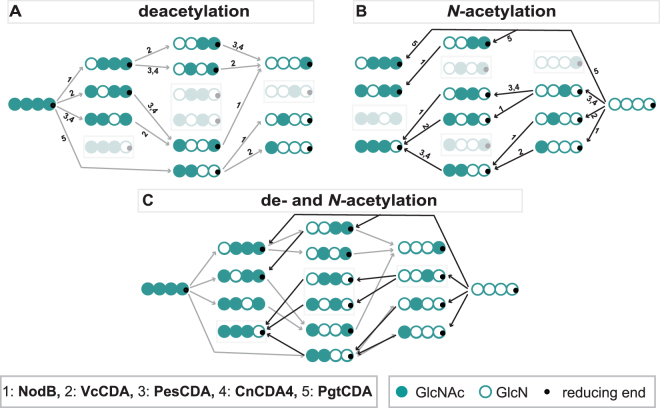
Production routes of all possible chitin and chitosan tetramers using CDAs to specifically deacetylate or *N-*acetylate (pa)COS; 1 represents the use of NodB, 2 of *Vc*CDA, 3 of *Pes*CDA, 4 of *Cn*CDA4, and 5 of *Pgt*CDA. Direction of routes are represented by arrows (grey: deacetylation, black: *N-*acetylation) and oligomers that are weak in color are not producible using this particular technique (de- or *N*-acetylation) (**A**) Production route of ten out of the 14 possible partially acetylated chitosan tetramers using different CDAs for deacetylation of the fully acetylated chitin tetramer as a substrate. (**B**) Production route of ten out of the 14 possible partially acetylated chitosan tetramers using different CDAs for *N-*acetylation under acetate-enriched conditions of the fully deacetylated chitosan tetramer as a substrate. (**C**) Overlay of production routes shown in part A (deacetylation) and B (*N*-acetylation), showing that all possible 14 partially acetylated chitosan tetramers are producible from the homotetramers A4 and D4.

After showing that bacterial as well as fungal CDAs can be combined with other CDAs from the same type of organism during *N-*acetylation, in a next step we investigated the possibility of combining fungal and bacterial CDAs with each other. As an example, the lower half of Fig. 5A shows the products generated by using the tetramer DDAD—produced by *Cn*CDA4 and *Pes*CDA—as substrate for *N*-acetylation by NodB, *Vc*CDA, or a combination of both. When added separately, NodB still efficiently acted on the non-reducing end (75%), and *Vc*CDA still *N-*acetylated the sugar unit next to the reducing end, as we have already described above (85%) (Figs 2 and 4). When both bacterial CDAs were combined, *N-*acetylation of DDAD was still very efficient and mainly resulted in the triple-acetylated paCOS with a PA of AAAD (80%). Figure 5B shows the efficient enzymatic production routes of four different, well-defined paCOS (DDAD, ADAD, DAAD, and AAAD). Importantly, none of these tetramers have, until now, been able to be produced in such a targeted way leading to highly PA-pure products —neither chemically nor enzymatically—as no CDA has been described as being able to specifically deacetylate the reducing end of COS. This fact points to the enormous potential for using CDAs to *N*-acetylate COS. By combining many different CDAs during deacetylation and *N*-acetylation, we managed, for the first time, to biotechnologically produce all of the 14 possible partially acetylated chitosan tetramers with high purity in DP, DA, and PA out of the pure chitin and glucosamine tetramer precursors. Figure 6A shows that not all paCOS can be produced by deacetylation alone, and Fig. 6B shows that not all paCOS can be produced by *N-*acetylation alone; but, by combining both techniques (Fig. 6C), all of the 14 possible partially acetylated chitosan tetramers can be produced with highly pure PAs.

However, as the enzymatic process of *N-*acetylation requires highly acetate-enriched conditions (2/2.5 M sodium acetate), rendering these well-defined paCOS unsuitable (in this state) for many applications and bioactivity tests that are needed to better understand the influence of PA on biological activities. To make these highly promising paCOS suitable for such tests, we first scaled up the production process and then developed a technique to purify them up to 95% purity.

### Purification of well-defined paCOS up to analytical grade

**Figure 7:**
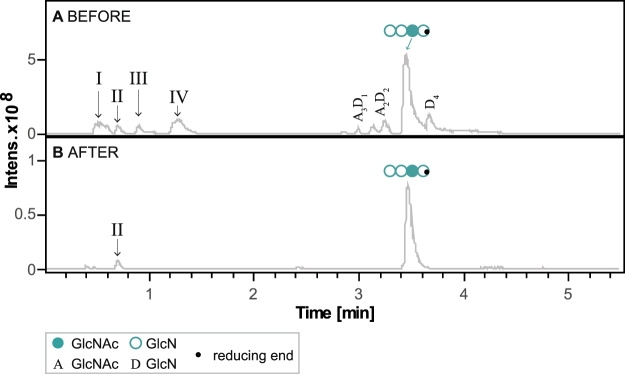
Chromatographic purification up to analytical grade of the (pa)COS DDAD. (**A**) Base peak chromatogram of HILIC-ESI-MS analysis showing both buffer components (I, II, III, IV) and (pa)COS available in the sample after enzymatic *N-*acetylation of the GlcN tetramer by *Pes*CDA and *Cn*CDA4 (A3D1, A2D2, A1D3. (**B**) Base peak chromatogram of HILIC-ESI-MS analysis showing both buffer components (II) and (pa)COS available in the sample after purification over HILIC, rotational vacuum concentration and lyophilization.

After scaling up the enzymatic production of the mono-acetylated chitosan tetramer (DDAD) by enzymatic *N-*acetylation to several milligrams, a method for purification was developed. About 1.5 to 2 mg of the pure chitosan tetramer was used as the substrate for *Cn*CDA4 and *Pes*CDA, and the reaction was performed as described before (acetate-enriched conditions, 24 h). We then analyzed the generated products as well as substances left over from the enzymes’ purification process, the *N-*acetylation-reaction, and buffer components (I-IV) by LC-MS (Fig. 7). Besides other components, such as d-Desthiobiotin (I), ammonium buffer (II), triethanolamin (III), and sodium acetate buffer components (IV), Fig. 7A shows the main-occurring paCOS products in the non-purified sample after enzymatic *N-*acetylation. In addition to the paCOS of interest, A_3_D_1_, we also found traces of D_4_, A_2_D_2_, and A_1_D_3_. To further use paCOS in, for example, bioactivity studies or applications, a high overall level of purity is required to ensure that one particular substance is responsible (or not responsible) for a particular activity. Furthermore, by working with substances of high overall purity, inhibition studies can be performed more confidently, such that a highly pure compound can be added to see if it inhibits the biological activity induced by a different highly pure compound.

To achieve the highest possible level of purity, the sample was purified via normal phase chromatography using a semi-preparative Luna CN (5 µm) column and a UHPLC system. Fractions of 1–5 ml were collected and analyzed using UHPLC-ELSD-ESI-MS analysis as previously described^11,35^. The fractions shown to contain the paCOS of interest (DDAD) were combined and further processed. After removing most volatile compounds like acetonitrile, the paCOS weight was determined and we were able to confirm that no measurable loss of sugars had occurred during the process (>95%). Figure 7B shows the LC-MS chromatogram of the mono-acetylated paCOS of interest DDAD after purification, and it indicates that except for the well-defined paCOS itself and a negligible amount of ammonium-buffer components, no impurities were present within the sample. Furthermore, because all other paCOS and other compounds present in the unpurified sample can be collected individually and either used, processed, or recycled, the whole purification process is highly valuable and economical. Combining classic enzymatic production of paCOS (deacetylation) with this newly discovered and highly regio-specific enzymatic method of *N-*acetylation, all of the 14 possible chitosan tetramers as well as many pentamers and hexamers can be produced and purified. Supplementary Figure S4 exemplarily shows LC-MS chromatograms of all possible mono-acetylated tetramers (ADDD; DADD; DDAD; DDDA) after purification which have either been produced by conventional enzymatic deacetylation of the GlcNAc tetramer or by enzymatic *N*-acetylation of the GlcN tetramer — thus using and combining different CDAs. This makes it possible to further analyze the influence of not only the DP and DA but also the PA on the biological activities of different chitosans. In addition, it is foreseeable that the chitosan products with defined and controllable PAs will be further modified in the future, as they possess highly reactive amino-groups; such next-generation products may lead us into a new era of chitosan research.

## Conclusion

In this study, we showed for the first time that a range of CDAs from different origins can efficiently *N-*acetylate chitosan oligomers, displaying the same regio-specificity that they possess during conventional deacetylation. Additionally, we showed that these CDAs also act on partially acetylated chitosan oligomers, again both in forward and reverse mode, allowing to combine different CDAs during both, deacetylation and *N-*acetylation. This enables us to produce all of the 14 possible partially acetylated chitosan tetramers and to further purify them to an analytical grade. The availability of these fully defined tetramers opens up new possibilities for the elucidation of structure-function relationships of chitosans, e.g. by studying their interaction with receptors or enzymes to elucidate these proteins’ subsite specificities or preferences.

## Electronic supplementary material

Supplementary Figures
