# Author Correction: Enzymatic production of all fourteen partially acetylated chitosan tetramers using different chitin deacetylases acting in forward or reverse mode

**DOI:** 10.1038/s41598-022-11538-5

**Published:** 2022-04-29

**Authors:** Lea Hembach, Stefan Cord-Landwehr, Bruno M. Moerschbacher

**Affiliations:** Institut für Biologie und Biotechnologie der Pflanzen, Westfälische Wilhelms-Universität Münster, Schlossplatz 8, 48143 Münster, Germany

Correction to: *Scientific Reports* 10.1038/s41598-017-17950-6, published online 18 December 2017

The Article contains an error in Figure 3, where the arrows in “*Pes*CDA” are incorrectly positioned.

**Figure 3:**
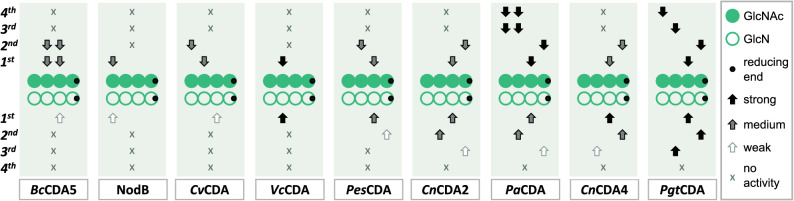
Mode of action of different CDAs (bacterial, viral, and fungal) on the chitin tetramer (A4, [GlcNAc]_4_, filled circles) shown in the upper part, as well as on the chitosan tetramer (D4, [GlcN]_4_, open circles) in the lower part. The reducing end of the (pa)COS always points to the right and is marked by a black circle. No activity is symbolized by an x, weak activity by an unfilled arrow, medium-strong activity by a grey-filled arrow, and strong activity by a black-filled arrow; arrows point to the unit of the (pa)COS which is preferentially de- or *N-*acetylated during either the 1st, 2nd, 3rd, or 4th attack by the different enzymes. Data summarized in this figure have been generated by (semi-)quantitative HILIC-ESI–MS analysis followed by ^18^O-labelling of the reducing end and MS/MS analysis.

The correct Figure 3 and accompanying legend appear below.

The original Article has been corrected.

