# Supplementary material for: Enzymatic production of all fourteen partially acetylated chitosan tetramers using different chitin deacetylases acting in forward or reverse mode
Source: Sci Rep. 2017 Dec 18;7:17692. doi: 10.1038/s41598-017-17950-6 (PMC5735187; doi:10.1038/s41598-017-17950-6)
Supplement: Supplementary file 1 — Supplementary Figures [file 41598_2017_17950_MOESM1_ESM.pdf]

Enzymatic production of all fourteen partially acetylated chitosan tetramers using different chitin deacetylases acting in forward or reverse mode

Lea Hembach[1], Stefan Cord-Landwehr[1], Bruno M. Moerschbacher[1,*]

[1]Institut für Biologie und Biotechnologie der Pflanzen, Westfälische Wilhelms-Universität Münster, Schlossplatz 8, 48143 Münster, Germany

* corresponding author

moersch@uni-muenster.de

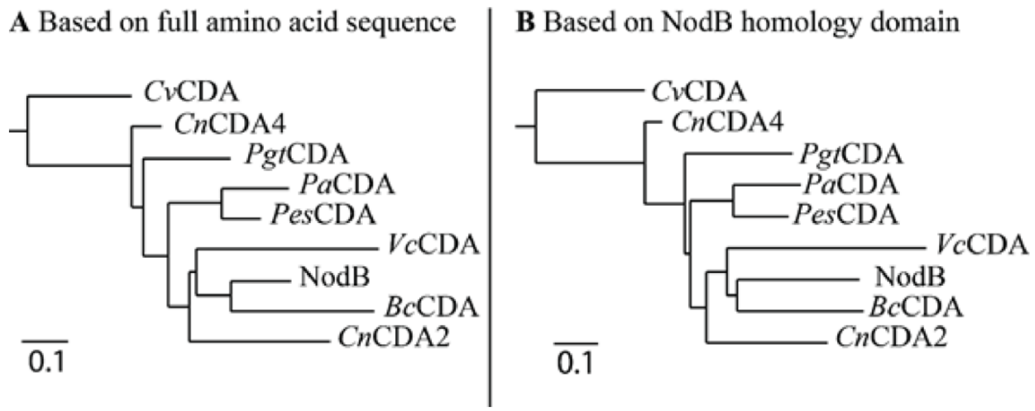

Supplementary Figure S1 Phylogram based on A) full amino acid sequence of CDAs and B) based on NodB homology domains generated using COBALT National center for biological information [44]. Branch length represents sequence similarity.

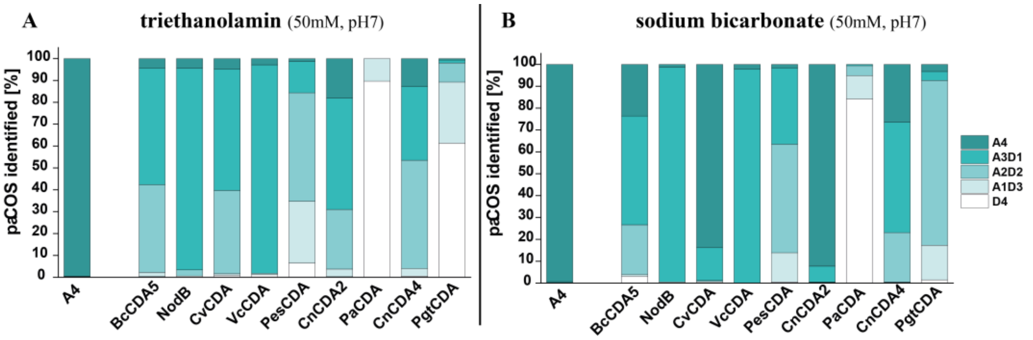

Supplementary Figure S2 Relative amounts of different chito-oligosaccharides after performing enzymatic deacetylation of the chitin tetramer (A4, [GlcNAc]$_{4,}$) using different CDAs [*Bc*CDA5, NodB, *Cv*CDA, *Vc*CDA, *Pes*CDA, *Cn*CDA2, *Pa*CDA, *Cn*CDA4, *Pgt*CDA] for 98 h in **A** 50mM triethanolamine buffer pH 7 and **B** 50mM sodium bicarbonate buffer, pH 7. Data summarized in this figure have been generated by semi-quantitative HILIC-ESI-MS analysis.

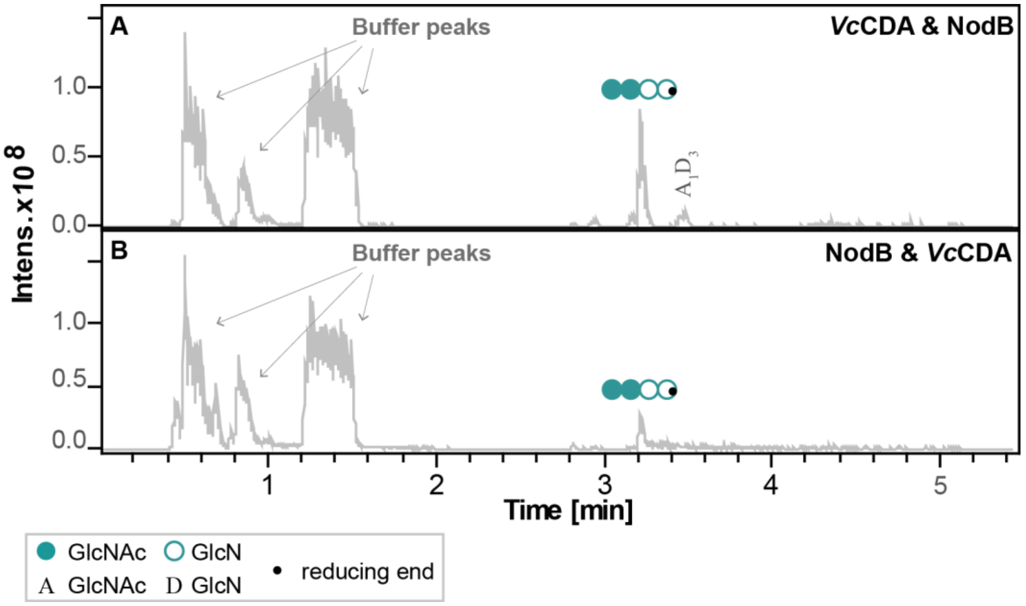

Supplementary Figure S3 Base peak chromatogram of HILIC-ESI-MS analysis showing products after *N*-acetylation of the chitosan tetramer (D4, $[GlcN]_4$, open circles) by two bacterial CDAs (VcCDA, NodB) with either A) first *Vc*CDA and in a second *N*-acetylation step NodB or B) first NodB and then *Vc*CDA.

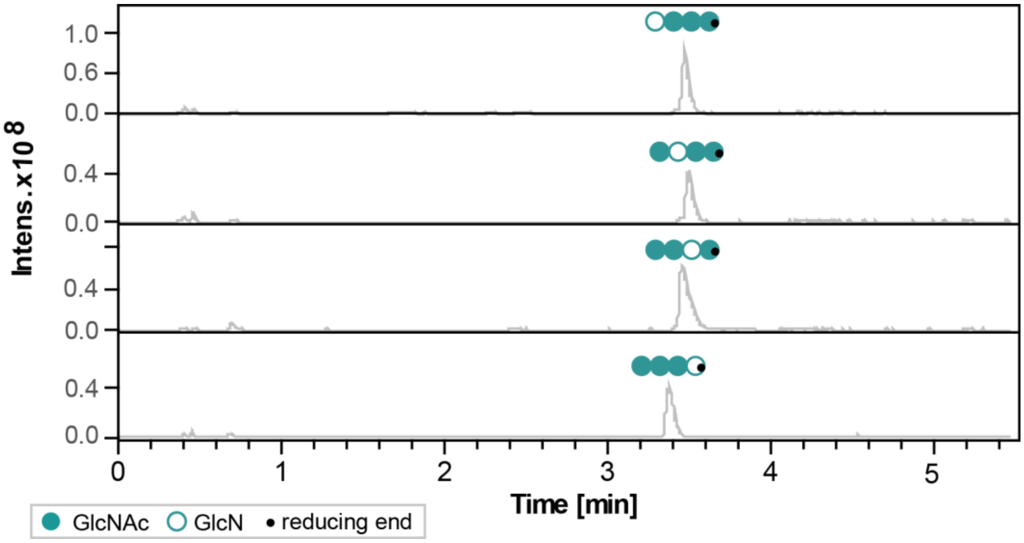

Supplementary Figure S4 Chromatographic purification up to analytical grade of different (pa)COS. Base peak chromatograms of HILIC-ESI-MS analysis showing purified A1D3 with different patterns of acetylation. Either produced by enzymatic deacetylation of the GlcNAc tetramer using *Vc*CDA in combination with *Pgt*CDA (ADDD), NodB in combination with *Pgt*CDA (DADD), NodB, *Vc*CDA and *Pes*CDA (DDDA) and enzymatic *N*-acetylation of the GlcN tetramer using *Pes*CDA and *Cn*CDA4 as shown in Figure 7.
